# Correction of Peri‐Implant Buccal Bone Dehiscence Following Sub‐Periosteal Peri‐Implant Augmented Layer Technique With Either Block or Particulate Xenograft: A Retrospective Study

**DOI:** 10.1111/clr.14400

**Published:** 2025-01-19

**Authors:** Mattia Severi, Franzini Chiara, Anna Simonelli, Chiara Scapoli, Leonardo Trombelli

**Affiliations:** ^1^ Research Centre for the Study of Periodontal and Peri‐Implant Diseases University of Ferrara Ferrara Italy; ^2^ Operative Unit of Dentistry Azienda Unità Sanitaria Locale (AUSL) Ferrara Italy; ^3^ Biology and Evolution Section University of Ferrara Ferrara Italy

**Keywords:** bone block, buccal bone dehiscence, implant placement, lateral bone augmentation, SPAL technique

## Abstract

**Objective:**

To evaluate the effectiveness of Sub‐periosteal Peri‐implant Augmented Layer (SPAL) technique performed with deproteinized bovine bone mineral (DBBM), delivered either as particulate (pDBBM) or block (bDBBM), in correcting a peri implant bone dehiscence (PIBD). Implants showing a thick (≥ 2 mm) peri‐implant buccal bone plate (PBBP) at placement were also examined.

**Material and Methods:**

Patients with a PIBD ≥ 1 mm, treated with SPAL with either pDBBM (SPAL_particulate_) or bDBBM (SPAL_block_), and patients with an implant showing a PBBP ≥ 2 mm at insertion (CONTROL) were included. Re‐entry was performed either at 6 months (SPAL groups) or 3 months (CONTROL). The rate of patients presenting no PIBD at re‐entry was the primary outcome. Bone dehiscence height (BDH) and width (BDW), thickness of buccal tissues (BTT) and marginal bone level (MBL) were secondary outcomes.

**Results:**

Thirty‐nine implants in 39 patients (14 in SPAL_particulate_,14 in SPAL_block_ and 11 in CONTROL) were analyzed. No PIBD were found in SPAL_particulate_ whereas in SPAL_block_ one PIBD was present. Two patients in CONTROL presented a PIBD. A reduction in both BDH and BDW was observed in both SPAL_particulate_ (2.7 ± 1.6 mm for BDH and 3.9 ± 0.2 mm for BDW) and SPAL_block_ (2.5 ± 1.8 mm for BDH and 3.8 ± 1.1 mm for BDW). SPAL_block_ showed a higher BTT than SPAL_particulate_ at re‐entry (3.6 ± 1.3 mm for SPAL_block_ and 2.6 ± 0.6 mm for SPAL_particulate_, *p* = 0.0160). All groups showed similar MBL.

**Conclusion:**

SPAL performed with either a pDBBM or bDBBM is similarly effective in correcting a PIBD as well as in increasing BTT.

## 1 Introduction

Prosthetically‐driven implant placement in a reduced horizontal bone dimension often results in a peri‐implant bone dehiscence (PIBD) (Bressan et al. 2017; Pramstraller et al. 2018). Small bony dehiscence defects left for spontaneous healing revealed more vertical bone loss at the buccal aspect after implant insertion and also more marginal bone loss compared to sites treated with GBR (Jung et al. 2017; Monje et al. 2023). Although implants with small, non‐contained buccal bone dehiscences exhibited high implant survival rates and healthy peri‐implant tissues at a 7.5‐year follow‐up (Waller et al. 2020), the presence of an overt PIBD was associated with a higher incidence of mucositis and peri‐implantitis as well as a faster progression of peri‐implantitis compared to implants completely surrounded by bone (Schwarz, Sahm, and Becker 2012; Monje et al. 2019). Despite the prognostic value of a PIBD on the long‐term peri‐implant tissue health is still controversial, the correction of a buccal bone dehiscence at implant placement has been recently recommended to favor the stability and healthy conditions of peri‐implant tissues over time (Herrera, et al. 2023; Monje et al. 2023; Song et al. 2024).

In order to treat a PIBD, either bone or soft tissue augmentation procedures were proposed. Both treatments were shown to provide stable clinical and radiographic outcomes in the medium and long‐term (Jensen et al. 2023; Monje et al. 2023). Among different lateral bone augmentation procedures at simultaneous implant placement, Guided Bone Regeneration (GBR) and Sub‐periosteal Peri‐implant Augmented Layer technique (SPAL, Trombelli et al. 2018) have been associated with a high probability of completely correcting a PIBD (Severi et al. 2022). In particular, SPAL is based on the use of patient periosteum as a barrier membrane to contain a graft acting as a “space‐making” scaffold. When performed in combination with a deproteinized bovine bone mineral (DBBM) particulate graft (pDBBM), SPAL was shown effective in completely correcting up to 90.9% of PIBDs (Trombelli et al. 2019, 2020). A recent retrospective study compared peri‐implant hard and soft tissue conditions at implants presenting either a PIBD treated with SPAL or a thick (≥ 2 mm) PBBP at implant placement (Trombelli et al. 2020). Although similar healthy conditions of the peri‐implant tissues were found in both groups, a more apical position of the radiographic marginal bone level (MBL) was found in SPAL group, suggesting a different remodeling pattern of the graft compared to native bone (Trombelli et al. 2020). These findings may question the relevance of the physico‐chemical characteristics of the graft with respect to the stability of the reconstructive outcome following SPAL. In this respect, an anectodotal report suggested that the use of a DBBM block (bDBBM) may be a successful alternative in combination with SPAL (Trombelli et al. 2022).

Therefore, the aim of the present retrospective study was to evaluate the effectiveness of SPAL performed in combination with either pDBBM or bDBBM in correcting a PIBD. Moreover, changes in the peri‐implant hard tissue occurring from implant placement to re‐entry for implant uncovering were analyzed. A control group comprising of implants showing a thick (≥ 2 mm) PBBP at implant placement was also examined.

## 2 Materials and Methods

### 2.1 Ethical Aspects

The present retrospective study was approved by the Ethical Committee of Area Vasta Emilia Centro, Italy (protocol n° 523/2024/Oss/UniFe, date of approval 16.10.2024). Each patient provided a written informed consent prior to surgical treatment. All the clinical procedures have been performed in accordance with the Declaration of Helsinki and the Good Clinical Practice Guidelines (GCPs).

### 2.2 Study Population

The record charts of patients undergone implant‐supported prosthetic rehabilitation in the period May 2020–September 2021 at the Research Centre for the Study of Periodontal and Peri‐implant Diseases, University of Ferrara, and at one private dental office based in Ferrara were screened to determine patient eligibility for the study. The present retrospective study was carried on following the STROBE statement guidelines/checklist for cross‐sectional studies.

Based on the conditions of buccal bone plate at the time of implant placement and surgical management, patients were categorized into 2 groups:

– Patients with at least one implant presenting a PIBD ≥ 1 mm, treated with SPAL in combination with pDBBM (SPAL_particulate_ group);
– Patients with at least one implant presenting a PIBD ≥ 1 mm, treated with SPAL in combination with bDBBM (SPAL_block_ group).

SPAL groups comprised of consecutively treated patients where pertinent clinical and radiographic measurements could be retrieved for data analysis. If two or more implants in the same patient were eligible for the study, only the implant presenting the PIBD with the highest bone dehiscence was selected for analysis. Moreover, patients with at least one implant presenting a residual PBBP thickness ≥ 2 mm after implant insertion were regarded as control group (CONTROL group).

All selected implants had to be placed in a healed bone crest (type 4C implants, Gallucci et al. 2018) and had to show primary stability, as assessed by insertion torque. Heavy smokers (cigarette consumption > 10 sigarette/day) and patients with diabetes mellitus at the time of surgery were excluded from the study.

### 2.3 Clinical Procedures

#### 2.3.1 Pre‐Operative Procedures

Prior to implant placement, all patients had undergone active therapy for treating carious lesions and periodontal diseases, and had been enrolled in a professional maintenance with frequency of recalls scheduled according to the PerioRisk assessment tool (Trombelli et al. 2009, 2017; Farina et al. 2021).

All the surgical procedures were performed by two trained operators (L.T., M.S.). Patients were administered 2 g of amoxicillin + clavulanic acid (Augmentin, GlaxoSmithKline, Verona, Italy) 1 h prior to surgery. Local anesthesia was attained using articaine with 1:100,000 epinephrine administered by local infiltration.

#### 2.3.2 Surgical Procedures

##### 2.3.2.1 SPAL Groups

In both SPAL_particulate_ and SPAL_block_ groups, surgical access to the bone crest was performed according to a previously described procedure (Trombelli et al. 2018). Briefly, a mucosal layer was raised on the buccal aspect by split‐thickness dissection with a 15C blade (Figures 1a and 2a). Then, the periosteal layer was elevated from the bone with a periosteal elevator (PTROM, Hu‐Friedy, Chicago, Illinois) as well as tunneling knives (KPAX, TKN1X and TKN2X, Hu Friedy, Chicago, Illinois) with varying angulated sharp edges, creating a sub‐periosteal pouch that could accommodate a graft (Figures 1b and 2b). A full‐thickness flap was elevated on the oral (lingual/palatal) aspect. Mylohyoid muscle fibers were detached from the lingual flap using a blunt instrument to allow for coronal advancement. Cortical perforations were performed with a calibrated cylindrical carbide bur in order to increase blood supply to the surgical area (Majzoub et al. 1999; Acar et al. 2016).

**FIGURE 1:**
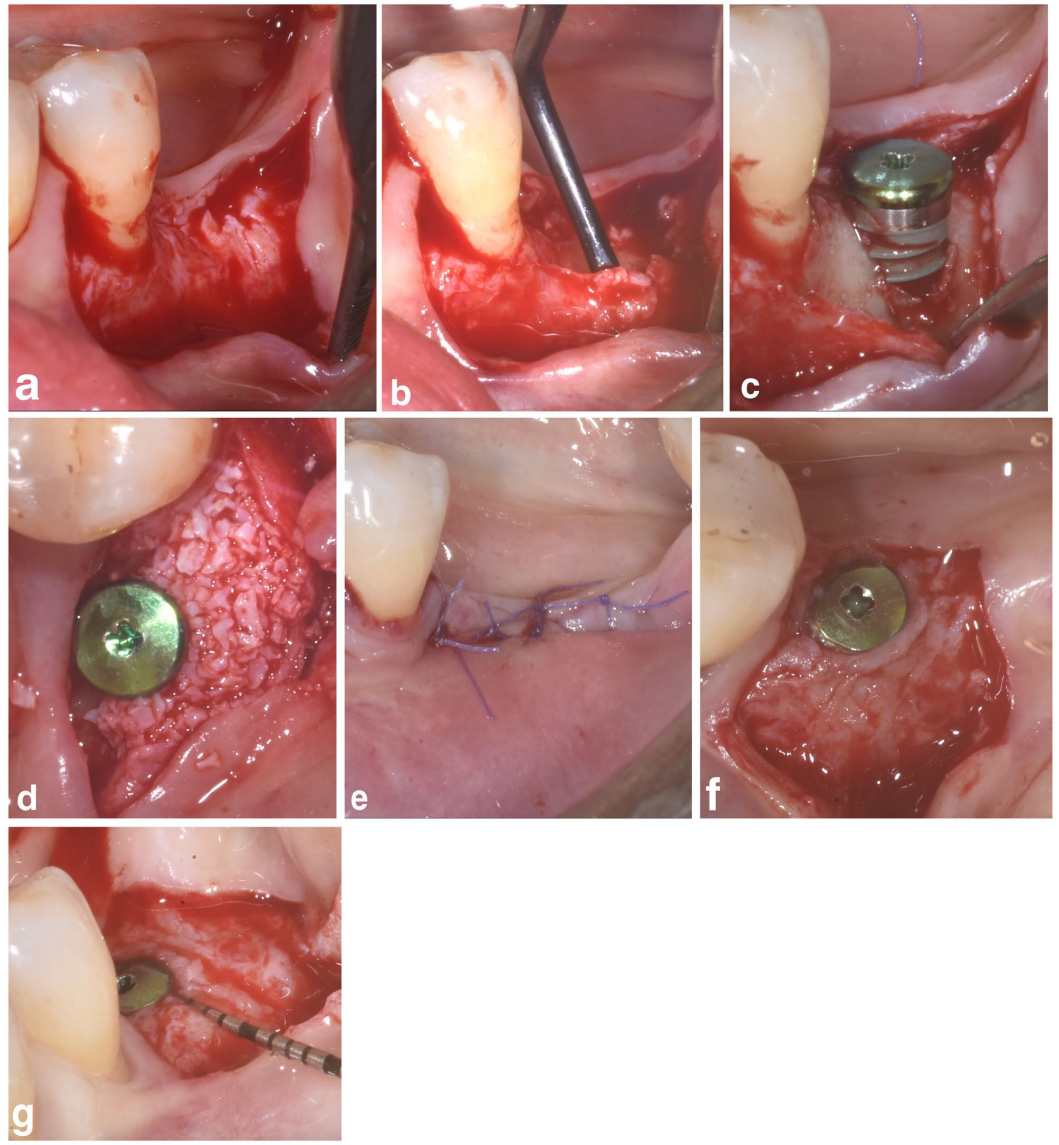
SPAL in combination with particulate DBBM (SPAL_particulate_ group). (a) The mucosal layer was raised on the buccal aspect by split‐thickness dissection. (b) The periosteal layer was elevated from the bone crest to create a sub‐periosteal envelope. (c) After placement, the implant showed a peri‐implant buccal bone dehiscence (PIBD). (d) Particulate DBBM was placed between the exposed implant surface and the periosteal layer to correct the PIBD and restore the missing buccal bone. (e) After suturing the periosteal layer to the lingual flap, mucosal layer was coronally advanced to cover both the graft and the implant. (f, g) At 6 months re‐entry, PIBD was completely corrected with a 3 mm thick buccal tissue.

**FIGURE 2:**
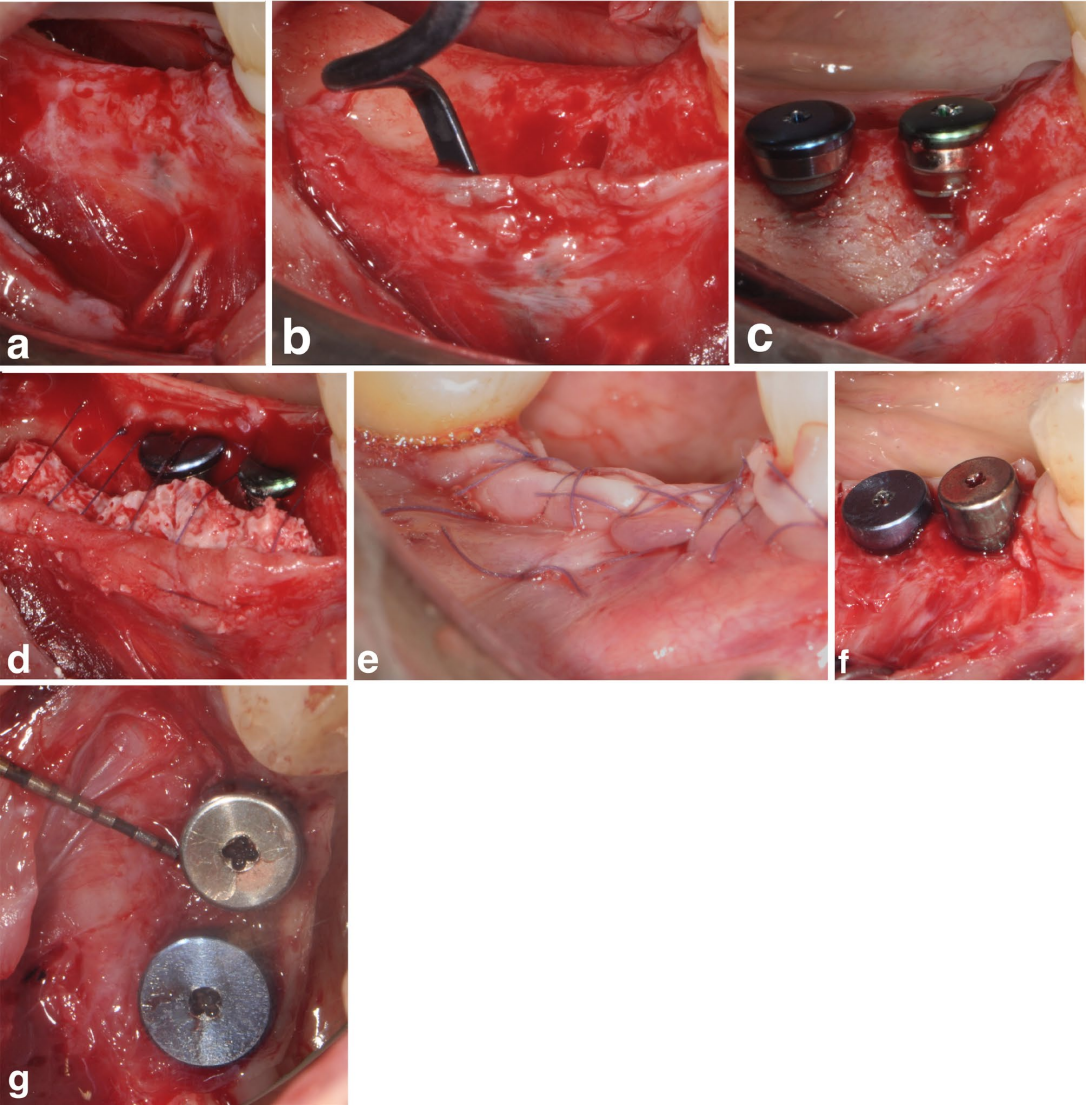
SPAL in combination with block DBBM (SPAL_block_ group) (a) The mucosal layer was raised on the buccal aspect by split‐thickness dissection. (b) The periosteal layer was elevated from the bone crest to create a sub‐periosteal envelope. (c) After placement, the implant in position 4.5 showed the with the highest bone dehiscence and then selected for the analysis. (d) DBBM block was trimmed and then stabilized between the exposed implant surface and the periosteal layer to correct the PIBD and restore the missing buccal bone. Periosteal layer was then sutured to the lingual flap. (e) Mucosal layer was coronally advanced to ensure primary closure. (f, g) At 6 months re‐entry PIBD were completely corrected with a 5 mm thick buccal tissue.

Tissue‐level implants (SPI Element; Thommen Medical, Grenchen, Switzerland) were placed with the coronal margin of the 1‐mm polished collar at the level of the bone crest (Figures 1c and 2c).

A bovine‐derived xenograft, delivered as either particulate (Bio‐Oss spongiosa granules, particle size 0.25–1.0 mm; Geistlich Pharma, AG, Wolhusen, Switzerland) (Trombelli et al. 2018) or block (Bio‐Oss block; Geistlich Pharma, AG, Wolhusen, Switzerland) (Trombelli et al. 2022), was used to fill the surgically‐created space between the periosteal layer and exposed implant surface. The block was fragmented into smaller pieces by means of a 15C blade or diamond bur in order to be adapted to the extent of the sub‐periosteal space and reach the desired buccal plate recontouring. Grafting was performed to completely correct the PIBD up to the bone crest. In all cases, the sub‐periosteal graft provided at least 2 mm of thickness at the polished collar of the implant.

The coronal portion of the periosteal layer was then secured to the oral flap by means of resorbable internal mattress sutures (Vicryl 6/0, Ethicon, Somerville NJ, USA) (Figures 1d and 2d). Subsequently, the mucosal layer was coronally advanced and sutured to the oral flap by horizontal internal mattress and interrupted sutures to provide a submerged healing for both graft and implant (Figures 1, 2).

At 6‐months surgical re‐entry for implant uncovering, a buccal split‐thickness flap was dissected to position the healing abutment. In order to assess the presence/absence of a residual PIBD, the presence of hard tissue around the implant surface was evaluated using a UNC‐15 probe. If no probe penetration beyond the polished implant collar was present, the implant was categorized as “completely corrected dehiscence”.

To provide adequate dimensions of keratinized peri‐implant mucosa (KT), either an apically positioning of the flap (APF) or a free gingival graft (FGG) was performed (Trombelli et al. 2019) (Figures 1f,g and 2f). The choice to perform either an APF or a FGG was based on the need to ensure a KT height of at least 2 mm at both buccal and lingual aspects of the implant.

##### 2.3.2.2 CONTROL Group

A buccal and lingual/palatal full thickness flap were raised to expose the bone crest. The implant site was prepared according to manufacturer instructions and tissue‐level implants (SPI Element; Thommen Medical, Grenchen, Switzerland) were inserted with the coronal margin of the 1‐mm polished collar at the level of the bone crest. No bone augmentation procedure was performed. In all cases, the flap was sutured using internal mattress and interrupted sutures (Vicryl 5/0, Ethicon, Somerville NJ, USA) to provide a submerged healing.

At 3‐months surgical re‐entry for implant uncovering, a buccal full‐thickness flap was elevated to screw the healing abutment. Flap was then trimmed and adapted to the healing abutment to provide adequate dimensions of peri‐implant keratinized mucosa.

##### 2.3.2.3 Postoperative Procedures

A rescue anti‐inflammatory drug (i.e., ibuprofen 600 mg tablets) was prescribed immediately after surgery, then *pro re nata* for the following postoperative days. Patients were instructed not to wear any removable prostheses to avoid compression onto the surgical site for at least 4 weeks, and not to chew or brush in the treated area for approximately 2 weeks. The home use of a 0.12% chlorhexidine solution (Dentosan, Recordati, Milan, Italy) was prescribed for chemical plaque control (1‐min rinse b.i.d. for 3 weeks). Sutures were removed at 2‐weeks post‐surgery.

### 2.4 Study Parameters

#### 2.4.1 Clinical Measurements

To evaluate the effect of the treatment on PIBD, the following clinical measurements were retrieved from clinical charts:

– Bone dehiscence height (BDH): measured at the mid‐buccal aspect of the implant as the distance between the apical margin of the polished collar of the implant and the first bone‐to‐implant contact;
– Bone dehiscence width (BDW): measured at the buccal aspect of the implant as the widest exposed portion of the rough implant surface.

In all groups, BDH and BDW had been assessed immediately after implant placement and at re‐entry for implant uncovering.

Moreover, the thickness of buccal tissues (BTT) had been measured as the distance between the buccal contour of the polished collar and the outer aspect of either the grafted area (SPAL groups) or native bone ridge (CONTROL group) at the mid‐buccal aspect of the implant. Baseline BTT had been recorded:

– Immediately after implant placement for the CONTROL group;
– Immediately after grafting for both SPAL groups;

BTT was recorded anew at surgical re‐entry for all groups.

All measurements had been performed using a UNC‐15 periodontal probe and rounded at the nearest millimeter.

#### 2.4.2 Radiographic Measurements

Periapical radiographs, taken with the long‐cone parallel technique immediately after surgery and at re‐entry for all groups, were digitized and analyzed using a specifically designed software (NIS elements v4.2; Nikon Instruments, Campi Bisenzio, Firenze, Italy). Marginal bone level (MBL) was measured as the distance (approximated to the nearest 0.1 mm) between the most apical margin of the polished implant collar and the bone crest at the mesial (mMBL) and distal (dMBL) aspect of each implant using a 10×–15× magnification. MBL was recorded as negative or positive when the apical margin of the polished collar was located either apically or coronally to the bone crest, respectively.

A reference mark 1‐mm high present on digital radiograph was used for calibration.

Two examiners (C.F. and M.S.) performed the radiographic measurements. Examiners were involved in a calibration session on a sample of radiographs obtained from patients not selected for the present study. The calibration session consisted of two sessions of MBL measurements, performed at a 7‐day interval, and allowed for reaching an excellent inter‐ and intra‐examiner agreement (*k*‐score 0.87).

## 3 Statistical Analysis

The rate of patients presenting the rough implant surface covered up to the apical part of the polished collar (i.e., no PIBD) at re‐entry was considered as the primary outcome. Values and changes in BDH, BDW, BTT and MBL were secondary outcome variables.

Sample size was calculated using data derived from a previous study (Benic et al. 2019) where the rate of patients presenting the rough implant surface covered up to the implant shoulder at re‐entry was 91.7% in DBBM block groups and 25.0% in DBBM particulate group, respectively. *Z*‐test estimated that at least 11 patients for each of the two independent SPAL groups were required to achieve 95% statistical power with an alpha error of 0.05.

The patient was regarded as the statistical unit. Data were described using mean, standard deviation, median, interquartile range (IR) and minimum‐maximum values for quantitative variables, and percentage proportions for categorical variables.

Intra‐group comparisons for continuous/ordinal variable were performed using the Wilcoxon signed‐rank test for paired data. Inter‐group comparisons for continuous/ordinal variable were performed using Kruskal‐Wallis ANOVA. In case of significance at Kruskal–Wallis test, multiple (post hoc) comparisons of average ranks have been computed; normal *z*‐values were computed for each comparison, as well as post hoc probabilities (corrected for the number of comparisons) for a two‐sided test of significance. Inter‐group comparisons for binary variable were performed using Maximum‐Likelihood Chi‐square tests with Yate's correction. The change in the secondary outcome variables was statistically evaluated using Generalized Linear Models (GLZ) adjusted for the significant confounders, followed by ANOVA for repeated measures for inter‐group comparisons and with post hoc unequal N HSD test.

## 4 Results

### 4.1 Study Population

Thirty‐nine implants in 39 patients (14 in SPAL_particulate_ group, 14 in SPAL_block_ group and 11 in CONTROL group) were selected for analysis (Table 1). No differences in age, gender and smoking status were observed among groups, the vast majority of the patients being non‐smokers. In SPAL_particulate_ and CONTROL groups one third of the implants were located in the maxilla, whereas all implants were located in the mandible in SPAL_block_ group. This difference was statistically significant (*p* = 0.027). Implants placed in SPAL_block_ group where significantly shorter than those placed in both SPAL_particulate_ and CONTROL groups (*p* = 0.008).

**TABLE 1:** Patient and implant characteristics in SPAL_particulate_, SPAL_block_ and CONTROL group. Categorical variables are described using count and percentage, and numerical variables are expressed as median, interquartile range (IR) and minimum‐maximum (min‐max) range.

	SPAL_particulate_ (14 patients)	SPAL_block_ (14 patients)	CONTROL (11 patients)	Inter‐group comparison
Patient characteristics				
Age (years)	63 (IR: 55–65.0; min‐max: 45.0–70.0)	55.0 (IR: 50–68.0; min‐max: 45.0–71.0)	46.0 (IR: 44.0–60.0; min‐max: 32.0–71.0)	0.081
Males/ Females	2/12	3/11	3/8	0.738
Smokers/non‐smokers	5/9	3/11	3/8	0.725
N° cigarettes/day (averaged only for smokers)	9	9.7	6.7	0.359
Implant characteristics				
Implant length (mm)	9.5 (IR: 9.5–9.5; min‐max: 8.0–11)	8.0 (IR: 8.0–9.5; min‐max: 6.5–9.5)	9.5 (IR: 8–9.5; min‐max: 8–9.5)	0.008^a^
Implant diameter (mm)	4.0 (IR: 4.0–4.0; min‐max: 3.5–4.2)	4.0 (IR: 4.0–4.2; min‐max: 3.5–4.2)	4.2 (IR: 4.0–4.2; min‐max: 3.5–4.2)	0.105
Implant position (maxilla/mandible)	5/9	0/14	4/7	0.027^b^

^a^ Post‐hoc probabilities of multiple comparisons for Kruskal–Wallis Test. SPAL_particulate_ vs. control: 0.999. SPAL_block_ vs. control: 0.161. SPAL_particulate_ vs. SPAL_block_: 0.022.

^b^ Post‐hoc probabilities of multiple comparisons ML Chi‐square. SPAL_particulate_ vs. control: 0.964. SPAL_block_ vs. control: 0.027. SPAL_particulate_ vs. SPAL_block_: 0.015.

#### 4.1.1 Post‐Operative Healing

In both SPAL_particulate_ and CONTROL groups, early healing was uneventful in all patients.

One patient in SPAL_block_ group experienced a post‐operative infection at 4 weeks after surgical intervention. The patient reported swelling and pain in the surgically treated area, and a fistula draining purulent exudate was evident (Figure 3a–f). After partial thickness flap elevation, the DBBM block graft was found embedded in granulation tissue and therefore removed.

**FIGURE 3:**
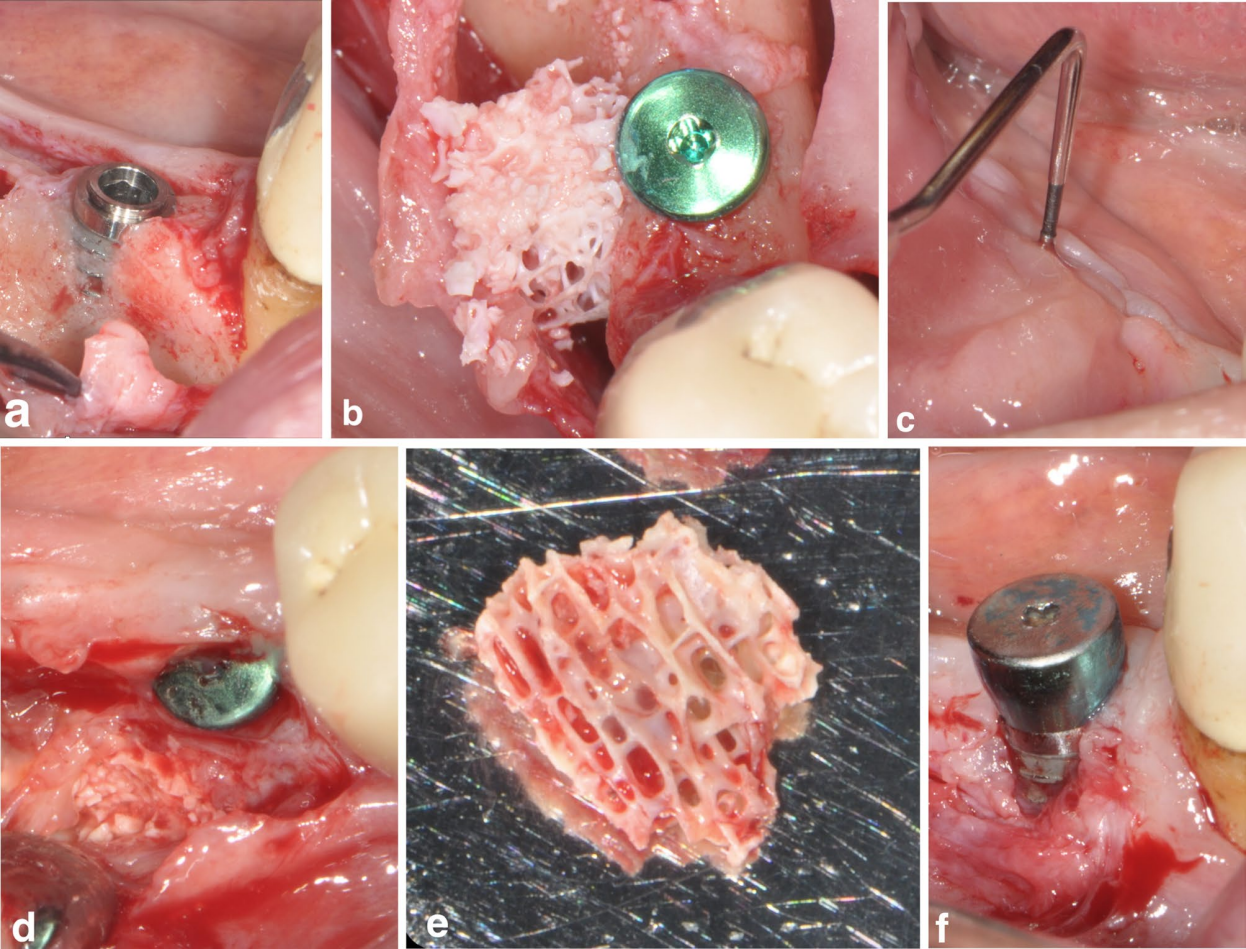
Post‐operative infection in one patient of the SPAL_block_ group. (a) Implant in position 4.5 presenting a PIBD at placement. (b) SPAL in combination with a DBBM block graft was used to correct the PIBD. (c) At 4 weeks post‐surgery, a fistula draining purulent exudate was present. (d, e) After flap elevation, DBBM block graft embedded in granulation tissue was completely removed. (f) A persistent PIBD was evident at 6‐months re‐entry.

### 4.2 Study Outcomes

#### 4.2.1 Primary Outcome

Number and % of patients presenting the rough implant surface covered up to the apical part of the polished collar at re‐entry (i.e., no PIBD) is shown in Table 2. No residual defect was found in SPAL_particulate_ group whereas in SPAL_block_ group the patient who had experienced the post‐operative graft infection presented a residual PIBD. Two patients in CONTROL group presented an incident PIBD at re‐entry (Table 2). The differences among groups were not statistically significant.

**TABLE 2:** Patients (number, %) presenting the rough implant surface covered up to the apical part of the polished collar at re‐entry (i.e., no PIBD) in SPAL_particulate_, SPAL_block_ and CONTROL group.

SPAL_particulate_ (*n* = 14)	SPAL_block_ (*n* = 14)	CONTROL (*n* = 11)	Inter‐group comparison
14 (100%)	13 (93%)	9 (82%)	0.336

#### 4.2.2 Secondary Outcomes

The GLZ model adjusted for the identified confounders (implant length and position) showed that both treatment and time were significant predictors for both BDH and BDW (time, *p* < 0.0001 for both; treatment, *p* < 0.001 for both). Within‐ and inter‐group comparisons for BDH and BDW are reported in Table 3. The Wilcoxon test for paired data showed a significant reduction in time for both SPAL_particulate_ (*p* < 0.001 for both BDH and BDW) and SPAL_block_ groups (*p* = 0.001 for both BDH and BDW). At implant placement, SPAL groups were both significantly different from CONTROL group (*p* = 0.0001 for both BDH and BDW). However, no significant differences were detected among groups at re‐entry for both BDH and BDW. In SPAL_block_ group the patient with the residual PIBD showed a BDH of 3 mm and a BDW of 4 mm. In CONTROL group the two patients with an incident PIBD at re‐entry showed a BDH of 2 mm and BDW of 2 mm and a BDH of 2 mm and BDW of 4 mm, respectively.

**TABLE 3:** Bone dehiscence height (BDH) and bone dehiscence width (BDW) in SPAL_particulate_, SPAL_block_ and CONTROL group.

Mean (SD)/Median (IQR; min‐max)	BDH			Within‐group comparison	BDW			Within‐group comparison
	Implant placement	Re‐entry	Change		Implant placement	Re‐entry	Change	
SPAL_particulate_ (14 patients)	2.7 (1.6)/2.0 (IR: 1.0–4.0; min‐max: 1.0–6.0)	0 (0)/0 (IR: 0–0; min‐max: 0–0)	−2.7 (1.6)/−2.0 (IR: −1.0 to −4.0; min‐max: −1.0 to −6.0)	< 0.001	3.9 (0.2)/4.0 (IR: 4.0–4.0; min‐max: 3.5–4.2)	0.0 (0.0)/0 (IR: 0–0; min‐max: 0–0.0)	−3.9 (0.2)/−4.0 (IR: −4.0 to −4.0; min‐max: −4.2 to −3.5	< 0.001
SPAL_block_ (14 patients)	2.6 (1.5)/2.0 (IR: 2.0–3.0; min‐max: 1–7)	0.2 (0.8)/0 (IR: 0–0; min‐max: 0–3)	−2.5 (1.8)/−2.0 (IR: −3.0 to −2.0; min‐max: 0 to −7)	0.001	4.1 (0.2)/4.0 (IR: 4.0–4.2; min‐max: 3.5–4.2)	0.3 (1.1)/0 (IR: 0–0; min‐max: 0–4)	−3.8 (1.1)/−4.0 (IR: −4.2 to −4.0; min‐max: 0 to −4.2)	0.001
CONTROL (11 patients)	0 (0)/0 (IR: 0–0; min‐max: 0–0)	0.4 (0.8)/0 (IR: 0–0; min‐max: 0–2)	0.4 (0.8)/0 (IR: 0–0; min‐max: 0–2)	0.999	0 (0)/0 (IR: 0–0; min‐max: 0–0)	0.2 (0.6)/0 (IR: 0–0; min‐max: 0–2)	0.5 (1.3)/0 (IR: 0–0; min‐max: 0–4)	0.999
Inter‐group comparison	< 0.0001	0.266	< 0.0001		< 0.0001	0.555	< 0.0001	
Post hoc unequal N HSD test	SPAL_particulate_ vs. control: 0.0001 SPAL_block_ vs. control: 0.0001 SPAL_particulate_ vs. SPAL_block_: 0.999		SPAL_particulate_ vs. control: 0.0001 SPAL_block_ vs. control: 0.0002 SPAL_particulate_ vs. SPAL_block_: 0.999		SPAL_particulate_ vs. control: 0.0001 SPAL_block_ vs. control: 0.0001 SPAL_particulate_ vs. SPAL_block_: 0.994		SPAL_particulate_ vs. control: 0.0006 SPAL_block_ vs. control: < 0.0001 SPAL_particulate_ vs. SPAL_block_: 0.999	

*Note:* Data are expressed in mm as mean, standard deviation (SD), median, interquartile range (IR) and minimum‐maximum (min‐max) range.

For BTT, the GLZ model adjusted for the identified confounders showed that both treatment and time were significant predictors (*p* < 0.0001 for both factors). Within‐ and inter‐group comparisons for BDH and BDW are reported in Table 4. A significant difference among groups was observed either at baseline and re‐entry (*p* < 0.0001). In particular, SPAL_block_ group showed a significantly greater values than SPAL_particulate_ either at baseline (*p* = 0.0022) and re‐entry (*p* = 0.0160). Within‐group comparisons showed a significant reduction in BTT over time for all groups, the magnitude of this reduction being similar among groups.

**TABLE 4:** Buccal tissue thickness (BTT) in SPAL_particulate_, SPAL_block_ and CONTROL group. Baseline BTT had been recorded immediately after implant placement for the CONTROL group, and immediately after grafting for both SPAL groups.

Mean (SD)/Median (IQR; min‐max)	BTT			Within‐group comparison
	Baseline	Re‐entry	Change	
SPAL_particulate_ (14 patients)	3.4 (0.6)/3.5 (IR: 3.0 to 4.0; min‐max: 2.0 to 4.0)	2.6 (0.6)/3.0 (IR: 2.0 to 3.0; min‐max: 2.0 to 4.0)	−0.8 (0.7)/−1.0 (IR: −1.0 to 0; min‐max: −2.0 to 0)	0.008
SPAL_block_ (14 patients)	4.6 (1.1)/4.0 (IR: 4.0 to 5.0; min‐max: 3 to 7)	3.6 (1.3)/4.0 (IR: 3.0 to 4.0; min‐max: 0 to 5)	−1.1 (1.6)/−1.0 (IR: −1.0 to 0; min‐max: −6 to 0)	0.012
CONTROL (11 patients)	2.3 (0.5)/2 (IR: 2.0 to 2.75; min‐max: 2 to 3)	1.2 (0.8)/1 (IR: 1.0 to 2.0; min‐max: 0 to 2)	−1.0 (0.8)/−1 (IR: −1.5 to −0.25; min‐max: −2 to −0)	0.012
Inter‐group comparison	< 0.0001	< 0.0001	0.783	
Post hoc unequal N HSD test	SPAL_particulate_ vs. control: 0.0055 SPAL_block_ vs. control: < 0.0001 SPAL_particulate_ vs. SPAL_block_: 0.0022	SPAL_particulate_ vs. control: 0.0007 SPAL_block_ vs. control: < 0.0001 SPAL_particulate_ vs. SPAL_block_: 0.0160		

*Note:* Data are expressed in mm as mean, standard deviation (SD), median, interquartile range (IR) and minimum‐maximum (min‐max) range.

All groups showed similar mMBL and dMBL values after surgery and at re‐entry as well as changes over time (Table 5).

**TABLE 5:** Inter‐group comparisons for marginal bone level, assessed as mesial (mMBL) and distal (dMBL) in SPAL_particulate_, SPAL_block_ and CONTROL group.

Mean (SD)/Median (IQR; min‐max)	mMBL			dMBL		
	After surgery	Re‐entry	Change	After surgery	Re‐entry	Change
SPAL_particulate_ (14 patients)	−1.0 (0.4)/−1.1 (IR: −1.3 to −0.7; min‐max: −1.8 to −0.5)	−0.6 (0.9)/−0.9 (IR: −1.3 to −0.4; min‐max: −1.4 to 1.3)	−0.5 (1)/−0.1 (IR: −1.5 to 0.4; min‐max: −2.3 to 0.5)	−0.8 (0.5)/−1.0 (IR: −1.1 to −0.4; min‐max: −1.6 to 0.3)	−0.2 (0.9)/−0.6 (IR: −1.0 to 0.5; min‐max: −1.3 to 1.3)	−0.6 (1.1)/−0.3 (IR: −1.5 to 0.1; min‐max: −2.4 to 1.3)
SPAL_block_ (14 patients)	−0.7 (0.8)/−0.9 (IR: −1.1 to 0.0; min‐max: −1.6 to 0.9)	−0.7 (0.6)/−0.8 (IR: −1.1 to −0.5; min‐max: −1.6 to 0.8)	0 (0.9)/0 (IR: −0.3 to 0.1; min‐max: −2 to 1.9)	−0.5 (0.5)/−0.7 (IR: −0.8 to −0.5; min‐max: −1 to 0.4)	−0.5 (0.6)/−0.6 (IR: −0.9 to −0.3; min‐max: −1.3 to 1.2)	0.1 (0.5)/0.1 (IR: −0.2 to 0.3; min‐max: −0.9 to 0.8)
CONTROL (11 patients)	−1.0 (0.4)/−1 (IR: −1.3 to −0.7; min‐max: −1.6 to −0.3)	−0.8 (0.6)/−0.8 (IR: −1.3 to −0.6; min‐max: −1.7–0.5)	−0.2 (0.7)/−0.2 (IR: −0.4 to 0.1; min‐max: −1.8 to 1.1)	−0.5 (0.5)/−0.7 (IR: −0.9 to −0.2; min‐max: −1.1 to 0.5)	−0.3 (0.5)/−0.5 (IR: −0.7 to 0.2; min‐max: −1.3 to 0.5)	−0.1 (0.4)/−0.2 (IR: −0.3 to 0.1; min‐max: −0.8 to 0.9)
Inter‐group comparison	0.411	0.854	0.695	0.116	0.747	0.099

*Note:* Radiographic measurements are expressed in mm as mean, standard deviation (SD), median, interquartile range (IR) and minimum‐maximum (min‐max) range.

## 5 Discussion

The aim of the present retrospective study was to evaluate the effectiveness of SPAL technique performed in combination with DBBM in form of either particulate or block to completely correct a PIBD at implant placement. SPAL groups were also compared with a group of patients showing a peri‐implant buccal bone plate of at least 2 mm following implant placement. The results showed that, irrespective of DBBM form, implants treated with SPAL technique showed a similarly high rate of patients showing a complete defect correction at 6 months re‐entry. SPAL_block_ group showed a significantly greater BTT at both baseline and re‐entry. However, the extent of graft/bone remodeling was similar among groups.

The choice of using the rate of complete dehiscence correction as primary outcome was due to the fact that an untreated or partially corrected PIBD may (i) favor the occurrence of a biological complication (Monje et al. 2019; Schwarz, Sahm, and Becker 2012) and (ii) lead to an increased interproximal peri‐implant bone loss (Jung et al. 2017). The CONTROL group was included in order to evaluate the extent of remodeling of the two graft biomaterials compared to the native bone.

The effectiveness of SPAL performed in combination with a pDBBM for the treatment of a PIBD was consistent with that reported in previous clinical studies where the same graft particulate was used, showing a rate of complete dehiscence coverage ranging from 80% to 91% (Trombelli et al. 2019, 2020). These findings could be partly ascribed to biological and technical aspects specific to the regenerative procedure. In SPAL technique, the periosteum layer represents a source of osteogenic cells which may (i) favorably contribute new bone formation (Ceccarelli, et al., 2016) and (ii) act as a neo‐angiogenic inductor thus providing the early vascularization of the DBBM graft (Nobuto et al. 2005). Furthermore, the creation of a secluded sub‐periosteal space may allow for a proper graft accommodation and stabilization at the most coronal portion of the implant (Trombelli et al. 2018, 2019, 2020). Graft stability to support the osteoconductive activity of the graft is also enhanced by means of internal mattress sutures that secure the coronal portion of the periosteal layer to the oral flap. Consistently, a network meta‐analysis comparing different treatment options to completely correct a PIBD showed that SPAL with a pDBBM has the highest probability of success among the included procedures (Severi et al. 2022).

Recently, the use of a bDBBM in combination with SPAL was proposed as a promising alternative to a particulate graft (Trombelli et al. 2022). bDBBM was suggested to act as an efficacious osteoconductive scaffold due to its mechanical properties that encompass a limited dislocation at flap manipulation as well as a higher dimensional stability (Benic et al. 2016; Benic et al. 2017; Mir‐Mari et al. 2016). The effectiveness of bDBBM is supported by our findings which showed a 93% rate of complete PIBD correction. Moreover, the use of bDBBM resulted in a greater BTT than pDBBM at both baseline and at re‐entry. These results are consistent with those stemming from a study where the combination of a bDBBM and a collagen membrane, simultaneously with implant placement, was shown to be superior to the combination of the same membrane and a pDBBM in complete defect resolution and restoration of a thick buccal plate (Benic et al. 2019). It should be, however, stressed that one patient receiving the block graft experienced a post‐surgery graft infection leading to a persisting dehiscence at 6‐months. Previous studies where a xenogenic bone block was used for a ridge augmentation procedure performed prior to implant placement reported a rate of graft infection and subsequent removal from 33% (Ortiz‐Vigón et al. 2017) to 40% (Schwarz et al. 2021) of the treated cases. Preclinical (Benic et al. 2016; Benic et al. 2017) and clinical (Laas, et al., 2020) studies showed that bDBBM was poorly colonized by novel bone when compared to pDBBM. The observed limited bone formation was related to an impaired vascularization of the block due to its macrostructure (Laass et al. 2020). Whether and to what extent the use of a bDBBM might represent a safe and efficacious alternative to pDBBM when combined to SPAL technique needs be further evaluated.

Our study design may provide preliminary insight on the extent of graft remodeling following SPAL procedures with different graft forms compared to bone remodeling after implant placement. Despite SPAL_block_ group showed a significantly greater BTT both after surgery and at re‐entry, the magnitude of graft remodeling was similar for the two grafts and compared to native bone. BTT changes in SPAL_block_ group were consistent with previous studies evaluating graft remodeling following GBR procedure performed using a bDBBM in combination with a collagen membrane (Benic et al. 2019). In contrast, the same study showed that the mean reduction in thickness for a pDBBM plus collagen membrane was two‐fold greater (2 mm) compared to our data. Differences in BTT changes observed in the two studies may be explained by varying graft remodeling dynamics as well as extent of graft displacement consequent to the specific regenerative procedure. Collectively, these observations suggest the need to over‐correct the BTT dimension to compensate the post‐surgical horizontal shrinking of the graft irrespectively of the bone augmentation procedures used.

In our material CONTROL group consisted of patients where a PBBP thickness of at least 2 mm was present after implant placement. This choice was based on previous studies showing that PBBP thickness correlates with the incidence of vertical bone loss at buccal plate following implant insertion (Spray et al. 2000) and the consequent need for bone augmentation procedures (Roccuzzo, Imber, and Jensen 2021). Interestingly, a mean reduction in BTT of 1 mm was also observed in CONTROL group which led to two patients (18%) experiencing an incident PIBD at re‐entry. Bone remodeling exceeded that reported in previous studies where the reduction in PBBP thickness following implant placement ranged from 0.3 (Merheb et al. 2017) to 0.4 mm (Cardaropoli, Lekholm, and Wennström 2006). Different factors, including 3D implant positioning (Nomiyama et al. 2022), methods for implant site preparation (Baggi et al. 2008; Peker Tekdal et al. 2016), implant design/surface (Linkevicius et al. 2020; Galindo‐Moreno et al. 2016; Camarda et al. 2021), anatomical location (anterior/posterior, mandible/maxilla) (Ghaly et al. 2023), thickness of the peri‐implant mucosa (Maia et al. 2015; Suárez‐López Del Amo et al. 2016), and prosthetic connection (Linkevicius et al. 2015) may have partly accounted for this discrepant observation.

In order to facilitate primary intention closure, tissue level implants had been placed with the coronal margin of the polished collar at the level of the bone crest. In this respect, an impact of implant positioning on the amount of PIBD correction as well as the extent of peri‐implant bone remodeling following implant insertion cannot be excluded (Saleh et al. 2018). Moreover, SPAL groups and CONTROL group were re‐entered for implant uncovering at different post‐surgical periods (6 months vs. 3 months). This difference may have variously impacted on tissue remodeling following implant insertion.

Peri‐implant marginal bone loss following implant installation may result from physiological remodeling due to mechanical and thermal trauma during implant site preparation. Interestingly, in our material a limited amount of graft/bone remodeling was similarly observed among groups and was consistent with previous studies where tissue‐level implants were either equi‐crestally placed (Saleh et al. 2018) or treated with SPAL plus pDBBM (Trombelli et al. 2020).

Some limitations of the present study should be considered when interpreting its findings. The choice of a pilot study with a retrospective design was based on the lack of specific clinical indications (i.e., patient/defect related factors) to combine SPAL technique with either DBBM particulate or DBBM block due to the limited, anecdotal evidence (Trombelli et al. 2022) supporting the use of DBBM block in combination with SPAL technique. A specific DBBM formulation was used according to the operator's preference. This unbiased selection of the graft used has resulted in no significant differences in terms of patient and defect characteristics. However, some factors, such as anatomical location and length of implants, were unevenly distributed among groups and, although controlled by the statistical analysis, may have biased the observed results. Since the included patients were consecutively treated during the routine clinical activity, a UNC 15 probe was the regular/standardized tool used to take all the clinical measurements, including PIBD dimensions. Other measurement methods, such as a caliper (Roccuzzo, Imber, and Jensen 2021) or a specifically‐designed device (Merheb et al. 2017), could have resulted in more appropriate recordings of dehiscence size and BBT.

In conclusion, our results seem to indicate that SPAL performed in combination with either a particulate or block DBBM graft, is similarly effective in correcting a PIBD as well as in increasing BTT at 6 months.

## Author Contributions

**Mattia Severi:** conceptualization, methodology, writing – original draft, writing – review and editing, data curation, investigation. **Franzini Chiara:** conceptualization, writing – original draft, writing – review and editing, data curation, investigation, methodology. **Anna Simonelli:** methodology, writing – review and editing, writing – original draft. **Chiara Scapoli:** methodology, writing – review and editing, data curation. **Leonardo Trombelli:** writing – original draft, writing – review and editing, investigation, conceptualization, funding acquisition, methodology, validation, project administration, supervision.

## Conflicts of Interest

Prof. Leonardo Trombelli received grants and lecture fees from Geistlich Biomaterials, Hu‐Friedy and Thommen Medical. Dr. Mattia Severi received lecture fees from Thommen Medical., Dr. Chiara Franzini, Dr. Anna Simonelli and Prof. Chiara Scapoli declare no conflict of interest.

## Data Availability

The data that support the findings of this study are available from the corresponding author upon reasonable request.
